# Surgical reconstruction of the ossicular chain with custom 3D printed ossicular prosthesis

**DOI:** 10.1186/s41205-017-0015-2

**Published:** 2017-07-27

**Authors:** Jeffrey D. Hirsch, Richard L. Vincent, David J. Eisenman

**Affiliations:** 1 Department of Diagnostic Radiology, University of Maryland School of Medicine, Baltimore, USA; 2 Department of Otorhinolaryngology – Head & Neck Surgery, University of Maryland School of Medicine, Baltimore, USA

**Keywords:** 3D printing, Ossicles, Ossicular prosthesis, Conductive hearing loss

## Abstract

**Background:**

Conductive hearing loss due to ossicular abnormalities occurs from many causes, including trauma, infection, cholesteatoma, surgery and congenital anomalies. Surgical reconstruction of the ossicular chain is a well-established procedure for repair of ossicular defects, but is still plagued by high failure rates. Underlying disease and proper sizing of prostheses are two challenges that lead to component failure. Three-dimensional (3D) printing has been used successfully to solve a number of medical prosthesis problems. Custom 3D printing an individualized ossicular prosthesis would be a potential solution for the wide range of anatomic variation encountered in the pathological middle ear, and could decrease the rate of post-operative prosthesis displacement by increasing the likelihood of a proper fit, in addition to decreasing surgical time.

In this study, the incus was removed from three formalin-fixed cadaveric human temporal bones with no macro- or microscopic evidence of pathology. Imaging of the cadaveric bone was obtained using a standard temporal bone CT protocol. A custom prosthesis for each cadaveric human temporal bone was designed using the Mimics Innovation Suite software (Materialise, Belgium) and fabricated on a Form2 3D printer (FormLabs, Somerville, Massachusetts). Four surgeons then performed insertion of each prosthesis into each middle ear, blinded to the bone from and for which each was designed. The surgeons were asked to match each prosthesis to its correct parent bone.

**Results:**

Each prosthesis had unique measurements. Each of the four surgeons was able to correctly match the prosthesis model to its intended temporal bone. The chances of this occurring randomly are 1:1296.

**Conclusions:**

A custom 3D printed ossicular prosthesis is a viable solution for conductive hearing loss due to ossicular chain defects. Commercially available CT scanners can detect significant anatomic differences in normal human middle ear ossicles. These differences can be accurately represented with current 3D printing technology and, more significantly, surgeons can detect these differences.

## Background

Conductive hearing loss due to ossicular abnormalities has many etiologies including trauma, infection, cholesteatoma, surgery to treat these diseases, and congenital anomalies. Surgical reconstruction of the ossicular chain is a well-established procedure for repair of ossicular defects, but is still plagued by high failure rates, with success rates in closing the air-bone gap to less than 20 dB ranging generally from 55%–75% [1–8]. Poor hearing results in many cases can be attributed to anatomical factors and persistence or recurrence of an underlying disease process, such as tympanic membrane retraction, middle ear atelectasis, fibrosis or mucosal pathology. However, none of these fully accounts for persisting air-bone gaps following ossiculoplasty [9, 10]. That these factors do not fully account for the failure rates is also implied by the fact that similar results are obtained with ossicular chain reconstruction following middle ear trauma, a situation in which most of those factors are not an issue [11, 12]. Some degree of hearing loss can be attributable to the design of current prostheses, which do not capture all of the mechanical advantages of the normal ossicular chain. Nevertheless, it is still likely that improper fit, due to both inaccurate size, angulation and position of the prosthesis, plays a significant role. In one series with long-term follow-up, more than 40% of failures were attributed to prosthesis or surgeon related errors [10] Proper intraoperative sizing of a prosthesis is challenging, and can be affected by limited exposure and variability in the anatomic relationships of the ossicular remnants to each other or to the tympanic membrane, as well as by post-operative changes during the healing process. In particular, the medial-lateral distances between ossicular remnants, the anterior-posterior offsets, and the position of and their relationship to the tympanic membrane or neo-tympanic membrane vary widely from patient to patient in the pathologic setting, [13] and are not always readily amenable to reconstruction with off the shelf prostheses.

Three-dimensional (3D) printing has been used for a wide variety of medical applications [14–16]. Custom 3D printing an individualized ossicular prosthesis would be a potential solution for the range of anatomic variation encountered in the pathological middle ear. Custom designed prostheses could decrease the rate of post-operative prosthesis displacement, and improve the hearing outcomes, by increasing the likelihood of a proper fit. Custom printed prostheses would minimize the need for intraoperative estimates of size, and would therefore also decrease surgical time, with resultant cost savings. However, it is not known if current technologies are suitable for application to the small anatomic variations found in the middle ear. The small size of the middle ear and its ossicles present challenges both for reliable image acquisition to provide accurate data for prosthesis design, and for printing of prostheses that faithfully reproduce the measured differences.

The present study is designed to answer three specific questions, as a predicate for proof of concept for development of custom 3D printed ossicular prostheses:

1. Can data from commercially available computed tomographic (CT) scanners be used to design a custom-made ossicular prosthesis that reflects normal variations in ossicular anatomy?
2. Can current 3D printing technology produce custom sized prostheses that reflect those normal variations?
3. Can otologic surgeons detect the differences in the resultant prostheses?

If each of these is answered in the affirmative, then development of a customized, 3D printed ossicular prosthesis should be feasible.

## Methods

### Cadaveric model

Three formalin-fixed cadaveric human temporal bones with no macro- or microscopic evidence of pathology were chosen. Working under a binocular operating microscope with conventional middle ear surgical instruments, a tympanomeatal flap was elevated and the incus was removed from each bone. The bones were labeled for identification with bicortical drill holes through the squamosa (one, two or three holes).

### Image acquisition, equipment and software

Imaging of the cadaver temporal bones was obtained using a standard CT protocol on a Brilliance CT 64 Channel (Philips Healthcare, Amsterdam, The Netherlands). Imaging parameters were as follows: slice thickness 0.67 mm with 0.33 mm overlap; tube rotation time 0.75 s; filter set to Detail; tube voltage 140 kVp and current 300 mAs; collumation 64 × 0.625; matrix 768; resolution set to HI; and scan field of view 200 mm. The printer for fabrication of the prostheses was a Form2 3D printer (FormLabs, Somerville, Massachusetts). The printer uses stereolithographic (SLA) technology on an optically cured resin. Print parameters were a layer thickness of 25 μm using the black photoreactive resin. Digital prosthesis design was accomplished with the Mimics Innovation Suite (Materialise, Belgium).

### Design

**Fig. 1:**
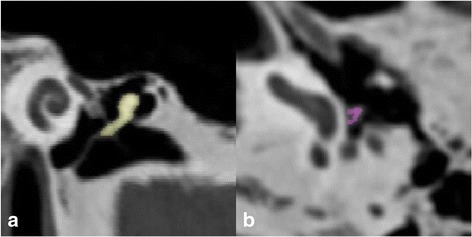
CT image-based masks. **a**. Mask of the malleus. **b**. Mask of the stapes

The prosthesis was designed to reestablish ossicular continuity following removal of the incus. The basic prosthesis design consisted of a trough for the manubrium of the malleus and a cup for the stapes capitulum, connected by a rigid columellar strut, mimicking a typical sculpted incus interposition graft, and similar mechanically to many prefabricated partial ossicular replacement prostheses (PORP’s) used in current practice. From CT imaging of the middle ear, a mask is created of the malleus and stapes, as seen in Fig. 1.

**Fig. 2:**
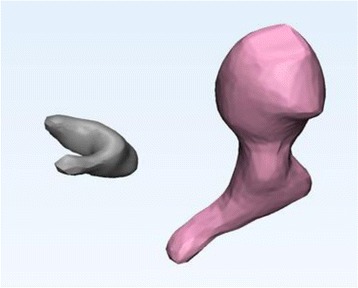
3D volume rendering of the malleus and stapes in correct anatomic position

The malleus is a relatively large and dense bone compared to the stapes. It is routinely well characterized by CT imaging and the creation of its mask is straightforward. The stapes is very small and not as radiodense. Fortunately, the capitulum and neck are the most radiodense part of the stapes and are usually well seen on CT. This is the most important part of the stapes to visualize, since it is the contact point for the prosthesis. The crura of the stapes are more gracile-shaped and usually faintly resolved by CT imaging, if at all. From the mask of the malleus and stapes a 3D shape is generated, shown in Fig. 2. Once these two landmarks are characterized the prosthesis can be designed.

**Fig. 3:**
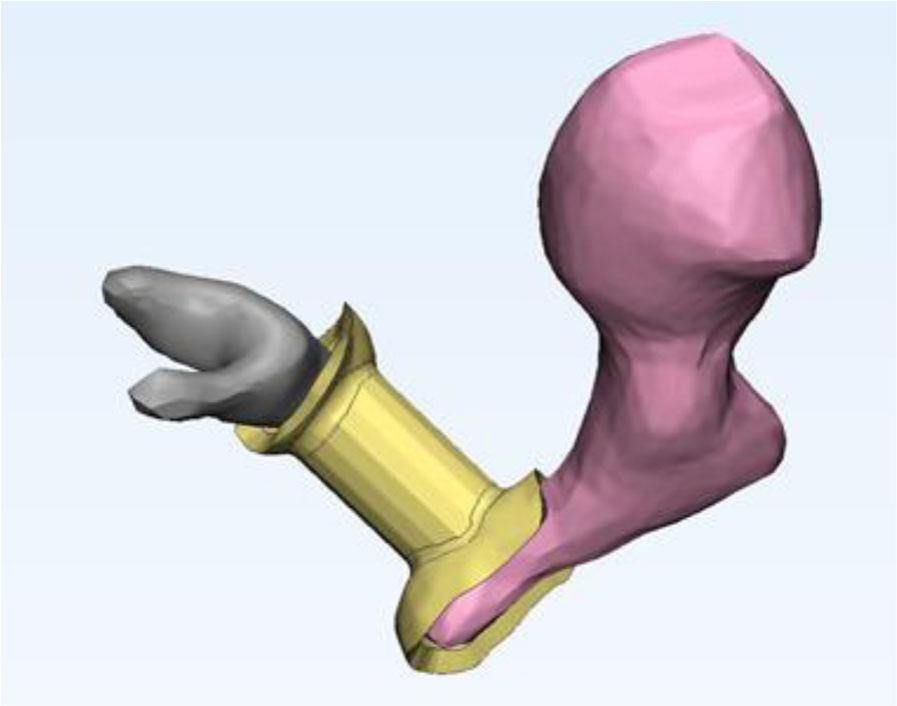
Design of the prosthesis in situ

The basic design of the prosthesis is a linear trough and cup on either end with a connecting strut, as shown in Fig. 3.

The linear trough will align with and fit over the manubrium of the malleus. The trough has a closed, curve-shaped end that fits over the distal end of the manubrium, the umbo. The cup fits over the stapes capitulum. The strut is a fabricated cylinder shape that connects the deep surface of the trough to the superficial surface of the cup.

### Measurements

**Fig. 4:**
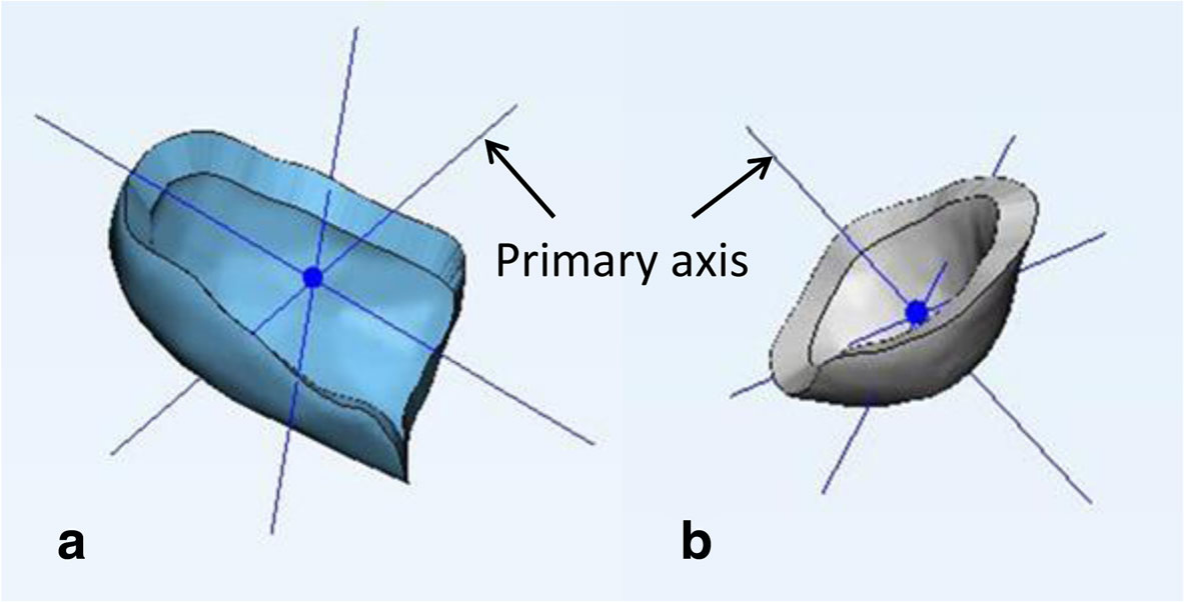
Mesh-based inertial axis of rotation. The inertial axis of rotation is shown with each shape; **a**. the trough and **b**. the cup. The primary axis is the line that is perpendicular to the face of the concave shape for each part

Accurately quantifying the model is important for understanding anatomic variations and establishing a format for reproducibility. The concept of inertial axis of rotation was used to establish a definition of position and angle of position for both the malleus trough and stapes cup, graphically represented in Fig. 4.

**Fig. 5:**
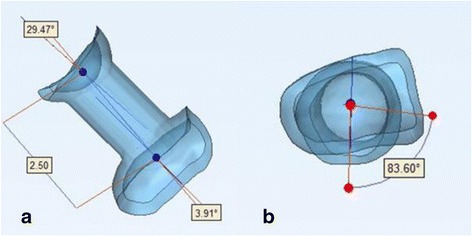
Defined prosthesis measurements. A schematic of the prosthesis for ear #1 showing the measurements defined by the prosthesis. **a**. The rotation from centerline and prosthesis length measurements. **b**. Rotation from trough to cup angle

Inertial axis of rotation is a mesh-based calculation of the modeled solid shape. The center of rotation and primary axis are calculated by the modeling software based on the unique design of the shape. Table 1 summarizes the quantifying data for the prosthesis of each cadaver middle ear. Figure 5 shows a schematic representation of the same data.

**Table 1:** Quantification data for each prosthesis. (Pr 1 – fabricated prosthesis for ear with one hole, Pr 2 – fabricated prosthesis for ear with two holes, Pr 3 – fabricated prosthesis for ear with three holes)

	Cup				Trough				Distance between centers of inertia (mm)	Rotation from cup to trough (degrees)
	Center of inertia			Rotation from centerline (degrees)	Center of inertia			Rotation from centerline (degrees)		
	X	Y	Z		X	Y	Z			
Pr 1	−1.33	218.03	333.56	29.47	−0.55	215.84	332.63	3.91	2.50	83.60
Pr 2	11.92	221.91	219.00	37.84	11.63	220.18	217.85	10.50	2.09	30.45
Pr 3	−4.09	207.58	101.18	21.28	−4.43	205.78	100.14	11.74	2.10	10.30

A line extending from the center of inertia defined by the malleus trough to the center of inertia defined by the stapes cup is the prosthesis length. The same line connecting the two centers of inertia is also used as a reference line to define the angular deviation or rotation from centerline of the primary axis of the malleus trough and stapes cup. Then, the angular rotation from the primary axis of the trough to the primary axis of the cup was also defined as the rotation of trough to cup.

### Blinded study

**Fig. 6:**
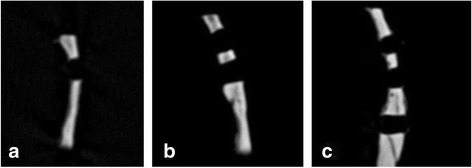
Identification of cadaveric temporal bones. Section of calvarium with identifying bicortical holes for each cadaver temporal bone; **a**. 1 hole, **b**. 2 holes, and **c**. 3 holes

Each cadaver middle ear was marked with a set number of bicortical drill holes (1 hole, 2 holes, or 3 holes) in a portion of the calvarium, as shown in Fig. 6. This made each bone unique and easy to differentiate both visually and by CT imaging.

**Fig. 7:**
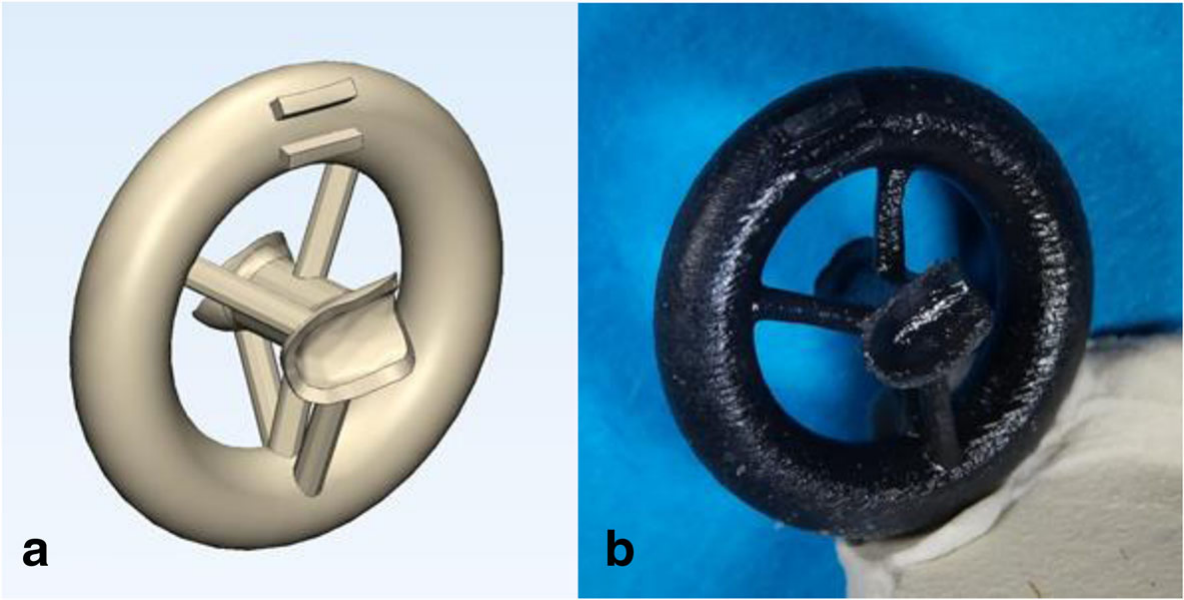
Prosthesis fabrication. **a**. STL file prior printing. **b**. Printed model for ear #1 with identifying “=” symbol embossed on the torus cradle

The model was fabricated from the CT imaging data from each cadaver middle ear. Each model was embossed with a symbol on the torus shape that cradled the prosthesis, as in the example seen in Fig. 7.

The symbols were an equal sign (=) for the cadaver temporal bone with one hole, a chevron (^) for the bone with two holes, and an asterisk (*) for the bone with three holes. Four surgeons- two attending physicians with practices limited to Otology and two chief residents both of whom had already completed senior level Otology rotations- then performed insertion of each prosthesis into each middle ear, blinded to the bone from and for which each was designed. The surgeons were asked to match each prosthesis to its correct parent bone.

## Results

### Difference in sizes of the prosthesis

By all quantitative measures, each prosthesis is unique. The lengths of the prosthesis between the respective centers of inertia ranged from 2.09 mm to 2.50 mm. The rotation from centerline of the trough ranged from 3.91 degrees to 11.74 degrees and the rotation from centerline of the cup from 21.28 degrees to 37.84 degrees. The rotation from the trough to the cup ranged from 10.30 degrees to 83.60 degrees.

### Surgical matching of each prosthesis

**Fig. 8:**
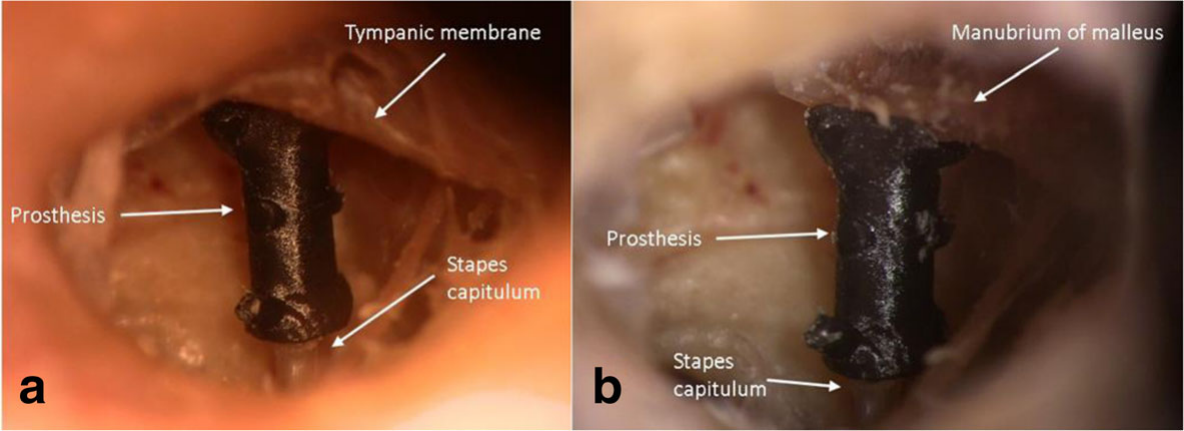
Prosthesis in situ. Prosthesis in situ in left middle ear, interposed between manubrium and capitulum. **a**. with tympanic membrane in place. **b**. with tympanic membrane removed

Four surgeons were asked to match the three prostheses, each with a unique identifying symbol, to the temporal bone with the best fit. The surgeons completed their task on separate days, blinded to the correct match. Each of the four surgeons was able to correctly match each prosthesis to its parent temporal bone. The chances of this occurring randomly are 1:1296. Photographic images of a prosthesis in place in the middle ear, both with the tympanic intact and with it removed, are shown in Fig. 8.

## Discussion

3D printed solutions have been shown to be successful adjuncts to surgical technique. Accurately reproducing a patient’s specific pathologic anatomy for preoperative planning is a common thread. Patient specific custom made anatomic models used in preoperative planning have been shown to decrease operative time [17] and in one report to also decrease intraoperative blood loss [18]. Additionally, models allowing for accurate surgical simulation in orthopedics and cardiovascular procedures have enhanced preoperative decision-making, improved precision and increased work efficiency [19, 20]. Prosthesis fabrication using 3D printed technique is another developing field [21, 22].

The present study demonstrates that 3D–printed ossicular replacement prostheses are unique in size and shape when using CT imaging as a basis for modeling, and that these differences are detectable by Otologic surgeons, who can accurately match the individual prostheses to their parent bones. The important landmarks within the middle ear are readily detectable during image interpretation of the middle ear. The malleus is usually well seen and masking of that bone is straightforward. The stapes is a much smaller bone and has a much smaller mass to attenuate the CT image beam. As a result the crura of the stapes, the thinnest part of the bone, are not well seen. However, the capitulum and neck of the stapes is more dense, and usually more reliably detected during image interpretation. This is important, because the stapes capitulum is where one side of the prosthesis rests. Once masks are made of these two landmarks a model can be designed and fabricated. The printer used to fabricate the prosthesis used SLA technology. The resolving threshold of the printer is on the order of centimicrons in the XY plane and decimicrons in the Z axis. This allows for an accurate representation of the model to be fabricated without significant intrinsic errors from the printer to be introduced to the prosthesis. The true test of accuracy, however, is if the prosthesis model fits in the space for which it was designed. In four separate trials with different surgeons, each surgeon was able to accurately match the correct prosthesis to its intended temporal bone. This further supports that the differences in size and shape for each prosthesis are meaningful and detectable by the surgeon. Additionally, that it is possible to fabricate a custom made middle ear prosthesis using routine CT imaging of the temporal bone, existing modeling software, and a desktop SLA printer.

Accurate quantification of the middle ear for fabrication of a custom prosthesis presented unique challenges. The process starts with identification of the important landmarks. As previously mentioned, the important landmarks for prosthesis construction are detectable with routine CT imaging protocols. The landmarks form the basis and starting point for fabrication of the prosthesis. As a result, subtle anatomic variation is inherently captured in the design of the prosthesis. Thus, establishing a method to accurately quantify parameters of the prosthesis also captures the subtle anatomic variation from ear to ear. Once the model was designed, then the next challenge is printing this very small part. Almost immediately apparent was the increased risk of losing the part during post processing due to its size. This was mitigated by utilizing a sinter box. The sinter box is a designed cage around a part that is fabricated with the part during the printing process. This, in essence, prints a larger part making it more difficult to lose. Additionally, it also provides a way to label the part and increases ease of handling. The torus cradle as seen in Fig. 7 is the sinter box used during printing of the prosthesis. Persisting conductive hearing loss following ossicular chain reconstruction is multifactorial. The single greatest variable in many cases is likely the underlying disease process, which may render the ear unsuitable for reconstruction over the long term [5, 9, 10]. Chronic infections and associated chronic Eustachian tube dysfunction can result in stiffness of the ossicular remnants, middle ear fibrosis, middle ear atelectasis, recurrent otitis media and other factors that decrease the chances of a satisfactory hearing result, either due to intrinsic limitations to adequate sound conduction, or from displacement and/or extrusion of the prosthesis. Nevertheless, technical factors such as imprecise sizing and placement also play a significant role [10]. These data are supported by the observation that outcomes are not significantly better, if at all, for reconstruction following traumatic ossicular discontinuity, [11, 12] a situation in which chronic infection and Eustachian tube dysfunction are not usually a factor. CT-based, custom 3D printed prostheses should minimize the impact of these variables, and consequently increase success rates.

Inability to accurately simulate the CT imaging in vivo is a technical limitation of the study. The cadaver middle ear used in this study was cut-down to size from the full-sized skull to include only a portion of the surrounding bone. As a result, the attenuation of the CT beam is much less for the cadaver ear and should provide a much better signal to noise ratio when compared to a comparable in vivo image data set. This will need to be addressed in future studies, as the important landmarks needed for prosthesis design are subtle imaging features that may be more challenging to detect on in vivo imaging.

An additional limitation of this study includes a lack of functional data. The design of the prosthesis should allow it to function similarly to existing, predicate models, and as such it would be presumed to result in adequate functional restoration of hearing. However, the present study does not offer comparative data, cadaveric or in vivo, demonstrating similar or better mechanical properties of the 3D reconstructed ossicular chain as compared to existing technologies. Another limitation of this approach, in general, is that it would only apply to clinical scenarios in which the middle ear anatomy will not otherwise be altered by the surgical procedure. If extensive removal of disease, including ossicular remnants, tympanic membrane, and/or portions of the external auditory canal and mastoid are planned, then pre-operative CT will not be able to predict the post-extirpative anatomy. This approach is only useful for patients undergoing a planned, isolated ossicular chain reconstruction, with no other procedural alterations in the anatomy.

Future studies will address these and other issues. Both cadaveric and in vivo functional results of a custom 3D printed prosthesis need to be measured, and compared to existing models. Additionally, the optimal biomaterial choice needs to be determined. Current materials have a high rate of extrusion when supported laterally by the tympanic membrane alone. As such, standard practice is to interpose a cartilage cap over the prosthesis to prevent that untoward outcome. This, however, can potentially dampen sound transmission, and adds another layer of risk for displacement of an element of the reconstruction. The ideal prosthesis would be fully biocompatible, and not require any additional protective layer.

## Conclusions

A custom 3D printed ossicular prosthesis is a viable solution for conductive hearing loss due to ossicular chain defects. Commercially available CT scanners can detect significant anatomic differences in normal human middle ear ossicles. These differences can be accurately represented with current 3D printing technology, and otologic surgeons can detect these differences in situ. This process overcomes the common technical challenge of properly sizing a prosthesis intraoperatively, as each model is custom made for an exact fit, and may lead to improved results and decreased operative time.

## Abbreviations

3D: Three-dimensional
CT: Computed tomographic
PORP: Partial ossicular replacement prosthesis
SLA: Stereolithographic

## References

[1] Berenholz LP, Burkey JM, Lippy WH (2011). Hearing results in reconstructing the damaged incus with varying lengths of the modified lippy prosthesis. Otol Neurotol.

[2] Fayad JN, Ursick J, Brackmann DE, Friedman RA (2014). Total ossiculoplasty: short- and long-term results using a titanium prosthesis with footplate shoe. Otol Neurotol.

[3] Hillman TA, Shelton C (2003). Ossicular chain reconstruction: titanium versus plastipore. Laryngoscope.

[4] Teufert KB, House JW (2001). Extrusion rates and hearing results in ossicular reconstruction. JAMA Otolaryngol Head Neck Surg.

[5] Mishiro Y, Sakagami M, Kitahara T, Kakutani C (2010). Prognostic factors of long-term outcomes after ossiculoplasty using multivariate analysis. Eur Arch Otorhinolaryngol.

[6] O'Connell BP, Rizk HG, Hutchinson T, Nguyen SA, Lambert PR (2016). Long-term outcomes of titanium Ossiculoplasty in chronic Otitis media. JAMA Otolaryngol Head Neck Surg.

[7] Quesnel S, Teissier N, Viala P, Couloigner V, Van Den Abbeele T (2010). Long term results of ossiculoplasties with partial and total titanium Vario Kurz prostheses in children. Int Pediatr Otorhinolaryngol.

[8] Truy E, Naiman AN, Pavillon C, Abedipour D, Lina-Granade G, Rabilloud M (2007). Hydroxyapatite versus titanium ossiculoplasty. Otol Neurotol.

[9] Dornhoffer JL, Gardner E (2001). Prognostic factors in ossiculoplasty: a statistical staging system. Otol Neurotol.

[10] Yung M (2006). Long-term results of ossiculoplasty: reasons for surgical failure. Otol Neurotol.

[11] Delrue S, Verhaert N, Dinther JV, Zarowski A, Somers T, Desloovere C (2016). Surgical management and hearing outcome of traumatic Ossicular injuries. J Int Adv Otol.

[12] Conoyer JM, Kaylie DM, Jackson CG (2007). Otologic surgery following ear trauma. JAMA Otolaryngol Head Neck Surg.

[13] Kaftan H, Bohme A, Martin H (2015). Geometric parameters of the ossicular chain as a function of its integrity: a micro-CT study in human temporal bones. Otol Neurotol.

[14] Chen X, Xu L, Wang W, Li X, Sun Y, Politis C (2016). Computer-aided design and manufacturing of surgical templates and their clinical applications: a review. Expert Rev Med Devices.

[15] Shafiee A, Atala A (2016). Printing Technologies for Medical Applications. Trends Mol Med.

[16] Tack P, Victor J, Gemmel P, Annemans L (2016). 3D-printing techniques in a medical setting: a systematic literature review. Biomed.

[17] Lethaus B, Poort L, Bockmann R, Smeets R, Tolba R, Kessler P (2012). Additive manufacturing for microvascular reconstruction of the mandible in 20 patients. J Craniomaxillofac Surg.

[18] Li C, Yang M, Xie Y, Chen Z, Wang C, Bai Y (2015). Application of the polystyrene model made by 3-D printing rapid prototyping technology for operation planning in revision lumbar discectomy. J Orthop Sci.

[19] Hughes AJ, DeBuitleir C, Soden P, O'Donnchadha B, Tansey A, Abdulkarim A, et al. 3D printing aids Acetabular reconstruction in complex revision hip Arthroplasty. Adv Orthop. 2017; doi:10.1155/2017/8925050. 10.1155/2017/8925050 PMC5259605 28168060

[20] Li H, Qingyao B, Shu M, Lizhong WX (2017). Application of 3D printing technology to left atrial appendage occlusion. Int J Cardiol.

[21] Kaye R, Goldstein T, Aronowitz D, Grande DA, Zeltsman D, Smith LP (2017). Ex vivo tracheomalacia model with 3D-printed external tracheal splint. Laryngoscope.

[22] Li H, Qu X, Mao Y, Dai K, Zhu Z (2016). Custom Acetabular cages offer stable fixation and improved hip scores for revision THA with severe bone defects. Clin Orthop Relat Res.

